# Homelessness and HIV Treatment Among Men Who Have Sex With Men Across US Funding Contexts

**DOI:** 10.1001/jamanetworkopen.2026.13609

**Published:** 2026-05-19

**Authors:** Yuanqi Mi, Stefan Baral, K. Alida Voet, Katherine Rucinski, John Mark Wiginton, Carrie Lyons, Kalai Willis, Tiara Willie, Travis Sanchez, Amrita Rao

**Affiliations:** 1 Department of Epidemiology, Johns Hopkins Bloomberg School of Public Health, Baltimore, Maryland; 2 Department of International Health, Johns Hopkins Bloomberg School of Public Health, Baltimore, Maryland; 3 Department of Mental Health, Johns Hopkins Bloomberg School of Public Health, Baltimore, Maryland; 4 Department of Epidemiology, Rollins School of Public Health, Emory University, Atlanta, Georgia

## Abstract

**Importance:**

Unstable housing has been shown to undermine health and exacerbate health inequities, including by limiting access to HIV prevention and treatment services among people living with HIV (PLHIV), the majority of whom are gay, bisexual, and other men who have sex with men (GBMSM). Assessing the outcomes of housing policies can inform evidence-based housing and homelessness prevention efforts in the US.

**Objective:**

To examine the association between homelessness and antiretroviral therapy (ART) use among GBMSM in the US and whether this association is moderated by the Housing Opportunities for People Living with HIV/AIDS (HOPWA), a federal housing program for PLHIV.

**Design, Setting, and Participants:**

This study was a cross-sectional analyses of 2017 to 2023 American Men’s Internet Survey (AMIS) data, a nationwide online survey. State-level HOPWA funding per person was estimated using collated secondary data. Participants were AMIS respondents who self-reported living with HIV and their treatment status.

**Exposure:**

Homelessness was self-reported as living on the street, in a shelter, in a single-room occupancy hotel, or in a car in the past 12 months.

**Main Outcomes and Measures:**

Current ART use among PLHIV was the primary outcome. Associations between homelessness and ART use were estimated using multilevel mixed-effects logistic regression, stratified by state-level HOPWA funding per person.

**Results:**

Among 58 108 AMIS respondents, 4911 (8.5%) self-reported living with HIV and were included in analyses (4709 [95.9%] were aged ≥25 years, 1339 [27.3%] were Black, 357 [7.3%] were Hispanic, and 1421 [28.9%] were White). Homelessness was associated with lower ART use (adjusted odds ratio [aOR], 0.51; 95% CI, 0.30-0.85). In states with at- or below-median HOPWA funding, homelessness was associated with decreased ART use (aOR, 0.43; 95% CI, 0.21-0.91). In higher-funded states, there was no association (aOR, 0.61; 95% CI, 0.30-1.27).

**Conclusions and Relevance:**

Homelessness is associated with reduced current ART use among GBMSM living with HIV. This association was not present in states with higher per-person HOPWA funding, suggesting that housing investments may mitigate barriers to HIV treatment. Taken together, these findings provide preliminary evidence of the positive health outcomes of HOPWA, underscoring the need for further research on how to optimize its effectiveness.

## Key Points

**Question:**

Is homelessness associated with lower current antiretroviral therapy (ART) use among gay, bisexual, and other men who have sex with men (GBMSM) living with HIV in the US, and is this association moderated by Housing Opportunities for Persons With HIV/AIDS (HOPWA), a federal housing program for people living with HIV?

**Findings:**

In this cross-sectional analysis of 2017 to 2023 AMIS data including 4911 GBMSM living with HIV, homelessness was associated with lower current ART use. This association was greater in states with at- or below-median HOPWA funding and appeared to be attenuated in states with above-median funding.

**Meaning:**

These findings suggest that higher per-person HOPWA funding may mitigate homelessness-related barriers to ART use among GBMSM living with HIV.

## Teaser

This cross-sectional study evaluates the association between homelessness and antiretroviral therapy use among gay, bisexual, and other men who have sex with men in the US.

## Introduction

Homelessness remains a pervasive social challenge in the US. According to the Department of Housing and Urban Development (HUD), more than 770 000 people experienced homelessness in 2024, representing the highest level ever recorded nationally.^1^ The housing affordability crisis, driven by rising rents and a shortage of accessible housing, is a primary driver of the growing rates of homelessness across the US.^1,2^ People living with HIV (PLHIV) are disproportionately affected by homelessness, as evidenced by a nationally representative study conducted in 2022 where nearly 9% of PLHIV reported homelessness, a prevalence roughly 50 times higher than that of the general population (0.18%) that same year.^3,4^ Housing instability has consistently been associated with negative outcomes across the HIV care continuum for PLHIV, including reduced HIV service use, greater HIV-associated morbidity and mortality, and challenges in achieving and maintaining viral suppression.^5,6,7^

Gay, bisexual, and other men who have sex with men (GBMSM) accounted for 71% of the 31 800 new HIV diagnoses in the US in 2022, and close to 20% of GBMSM reported unstable housing in that same year.^8^ Housing instability among GBMSM in the US is exacerbated by economic inequities. GBMSM earn approximately 10% to 32% less than their heterosexual counterparts in comparable positions, placing them at greater risk for housing insecurity.^9^ Unstable housing undermines antiretroviral therapy (ART) initiation and retention by creating practical barriers, such as transportation limitations (limited transportation funds and frequent moves and/or shelter turnover create difficulties traveling to clinic or health care facilities), poor health insurance coverage (applications and/or renewals typically require a mailing address), a lack of a private or secure space to store medications, and limited access to communication technologies (phone or internet) needed to schedule and keep medical appointments.^6,10,11^ These barriers likely contribute to gaps along the HIV care continuum. In 2022, among GBMSM living with HIV, only 78% accessed HIV treatment and 55% were retained in treatment, with an estimated 68% achieving viral suppression.^8^ Characterizing and ultimately addressing housing-related barriers may help partially close these gaps.

To address the intersecting crises of HIV and homelessness, HUD established the Housing Opportunities for Persons with HIV/AIDS (HOPWA) program through the National Affordable Housing Act of 1990 to address the housing needs of PLHIV.^12^ Funding for supportive housing programs, including HOPWA, was preserved in the 2026 federal budget. This is aligned with the data that the number of people experiencing homelessness continues to grow nationwide, increasing by 18% between 2023 and 2024.^13^ Eligibility for HOPWA grants is determined by a formula that incorporates local HIV burden, poverty rates, and fair market rents.^14,15^ However, per-person allocations and service capacity continue to vary markedly across states.^16^ While homelessness has been recognized as a social determinant of various health outcomes,^17^ its specific association with ART use and how differences in HOPWA funding modify the association has not been comprehensively studied among GBMSM in the US.

The objective of this study was to examine the association between homelessness and current ART use among GBMSM in the US and whether this association is moderated by a federal program designed to address the housing needs of PLHIV. Findings are intended to provide insights into the specific social and structural challenges faced by GBMSM living with HIV under varying policy and funding contexts.

## Methods

This cross-sectional study was conducted in compliance with federal regulations governing the protection of human participants and was reviewed and approved by the institutional review boards of Emory University and Johns Hopkins University. Participants provided written informed consent. The study adhered to Strengthening the Reporting of Observational Studies in Epidemiology (STROBE) reporting guidelines for cross-sectional studies.^18^

### Study Design and Setting

The American Men’s Internet Survey (AMIS) is an annual cross-sectional survey of cisgender GBMSM in the US. Recruitment is conducted online with the overall goal of monitoring trends in HIV risk behaviors and use of prevention and treatment services to support national, state, and local public health action.^19^ Recruitment for AMIS is conducted using convenience sampling that has been documented previously.^20^ Briefly, members of the target population are invited to participate via banner and message-based advertisements on online social networking websites and apps and other websites. Participants who either clicked on the ads or accessed the survey via emailed links were immediately directed to a secure server-hosted survey screener on Alchemer.^19^ Data used for the present analysis represent survey data collected from 2017 to 2023.

### Study Population

Individuals were eligible to participate in AMIS if they were assigned male sex at birth, currently identified as a male, were at least 15 years of age, had oral or anal sex with another man in their lifetime or self-identified as gay or bisexual (for those aged 15-17 years), and provided a valid US zip code.^21^ A total of 58 108 individuals participated in AMIS between 2017 and 2023, of whom 53 143 (91.5%) self-reported not living with HIV and were excluded from these analyses. Those that did not report current HIV treatment status (54 respondents [1.1%]) were excluded from these analyses.

### Study Procedures and Outcome

The survey included a series of questions related to demographic characteristics; sexual behaviors; HIV/STI testing history and diagnoses; drug and alcohol use; mental health; stigma related to sexual orientation; social support; and knowledge, interest, and use of specific HIV prevention and treatment modalities. The primary outcome was current use of ART, ascertained by asking, “Are you currently taking antiretroviral medicines to treat your HIV infection?” Participants could respond either “Yes” or “No.”

### Primary Independent Variable

The primary independent variable was homelessness, ascertained by asking the question, “In the past 12 months, were you ever homeless? That is, were you living on the street, in a shelter, in a single-room occupancy hotel (SRO), or in a car?”^22^ This definition of homelessness is aligned with the US Centers for Disease Control and Prevention (CDC) definition and is how homelessness was captured in the National HIV Behavioral Surveillance Survey.^23^

### Other Covariates

Participant characteristics were self-reported and included age, highest level of education completed, race and ethnicity, region, health insurance, year of recruitment, poverty based on household income, lifetime injection drug use, and HIV status. Age was categorized as binary: 15 to 24 years and 25 or more years. Highest level of education was categorized as high school or less vs some college or associate’s degree or more. Self-reported race and ethnicity was categorized based on classification from the US Census Bureau as Black, Hispanic, White, and other (including American Indian or Alaska Native, Asian, Native Hawaiian or Other Pacific Islander, and multiracial).^24^ Categories were restricted based on how questions were asked in the survey across all years. Year of recruitment referred to the year participants completed the survey. Region was categorized as Northeast, Midwest, South, West, and US Dependent Areas.^25^ Health insurance was defined as insured (any coverage) vs uninsured at the time of the survey. Household income was based on the question, “What was your household income last year from all sources before taxes?” Poverty was defined as income less than $40 000, which is consistent with 2024 US Census Bureau classifications for a family of 4.^26^ Lifetime injection drug use was assessed by asking, “Have you ever in your life shot up or injected any drugs other than those prescribed for you? By shooting up, we mean anytime you might have used drugs with a needle, either by mainlining, skin popping, or muscling?” HIV status was based on the question, “Did you ever test positive for HIV?” with response options “No,” “Yes,” “Prefer not to answer,” and “Don’t know.”

### State-Level Covariates

The Ryan White HIV/AIDS Program provides HIV care, treatment, and supportive services for PLHIV in the US.^27^ State-level Part B funding refers to annual grants awarded to each state.^28^ We calculated mean Part B funding per state from 2017 to 2023 and included it as a continuous state-level covariate.

### Effect Measure Modifier

State-level HOPWA funding per PLHIV experiencing homelessness was estimated across multiple years to correspond to the 2017 to 2023 recruitment period of AMIS participants, thereby better capturing the funding context relevant to participants’ exposure at enrollment. We used 3 sources of data (1) HOPWA data, (2) point-in-time (PIT) counts, and (3) state HIV prevalence. For HOPWA funding data, we obtained annual state allocations of HOPWA grant funding from the HUD Office of HIV/AIDS Housing for each state from 2017 through 2022 (data in 2023 not available).^12^ We calculated the mean funding allocated across these 6 years for each state. For PIT homelessness count, using HUD’s Annual Homeless Assessment Report data, we extracted the number of people experiencing homelessness in each state, based on PIT estimates from January of each year, 2017 to 2023.^29^ We calculated the mean number of people experiencing homelessness across these 7 years for each state. For state HIV prevalence, we obtained state-level adult HIV prevalence estimates for each year from 2018 to 2022 (data in 2017 and 2023 not available) from the CDC HIV Surveillance Report. We calculated the mean HIV prevalence across these 5 years for each state.^30^

State-level HOPWA funding per PLHIV experiencing homelessness was then calculated based on the following formula: (mean HOPWA funding)/[(mean number of people experiencing homelessness) × (mean HIV prevalence)]. This variable, which will be referred to as state-level HOPWA funding from here on, was used to categorize states into 2 groups: at or below median level of funding and above median level of funding.

Means were calculated for auxiliary data across several years to as closely align to years of survey data collection as possible. Because state-level counts of people simultaneously experiencing homelessness and living with HIV are not directly available, comparative proxy of funding intensity was constructed by combining publicly available homelessness counts and HIV prevalence, and used this measure solely for relative stratification across states. State-level data are presented in eTable 1 in Supplement 1.

### Statistical Analyses

Descriptive statistics of participant sociodemographic and behavioral characteristics are presented overall and by experience of homelessness. Differences between those who did and did not experience homelessness were tested using χ^2^ tests.

Individual-level variables were nested within states; this hierarchical structure was accounted for using mixed-effects logistic regression models with random intercepts for states. Mixed-effects logistic regression with random intercepts for states was used to estimate the association between homelessness and current ART use, adjusting for state-level Ryan White Part B funding. A multivariable mixed-effects logistic regression model with random intercepts for states was used further adjusting for age, injection drug use, education, race, year of recruitment, health insurance, and poverty. Confounders were selected using a directed acyclic graph (DAG), and multicollinearity was assessed and found to be minimal. We included random intercepts only because the primary objective was to adjust for between-state differences in baseline ART use. Intraclass correlation coefficients (ICCs) were used to quantify between-state variability. To assess effect measure modification on the multiplicative scale, analyses were stratified by above the median (high) vs at or below the median (low) state-level HOPWA funding. Estimated probabilities from the adjusted mixed-effects logistic regression models overall and after stratification were calculated to contextualize the magnitude of the association. We examined the distribution of ART use by homelessness status, overall and stratified by HOPWA funding, to demonstrate no quasicomplete separation (eTable 4 in Supplement 1). A *P* value less than .05 was considered significant. Analyses were conducted in R Studio version 4.2.1 (R Project for Statistical Computing). As missing data were less than 2% for all variables, available case analysis was used.

We conducted a sensitivity analysis of the specification of the effect measure modifier by using state-level HOPWA funding based on 2022 data alone (most recent data available) rather than a mean across 2017 to 2022. Additionally, to assess whether alternative modeling approaches led to the same inferences, we conducted a sensitivity analysis using linear probability models (LPMs) with robust standard errors clustered by state to estimate prevalence differences.

## Results

Overall, 388 of 4911 GBMSM living with HIV (8.2%) reported experiencing homelessness in the 12 months preceding data collection, and 4760 (96.9%) participants reported current ART use. Most participants were aged 25 years or older (4709 participants [95.9%]) and reported having a college degree, associate’s degree, or higher (4239 participants [88.3%]). Approximately 30% of participants were Black (1339 [27.3%] were Black, 357 [7.3%] were Hispanic, and 1421 [28.9%] were White). Close to 40% of participants were living in poverty (1737 participants [37.7%]), and about half reported residing in the Southern US (2350 participants [47.9%]) at the time of data collection. Most participants had some form of health insurance (4668 participants [95.1%]). Close to 1 in 5 reported injecting drugs during their lifetime (883 participants [18.2%]). Just over half of the participants were from states that had low levels of HOPWA funding per person (2724 participants [55.6%]).

There were significant differences by experiences of homelessness across most of the included covariates, with the exception of age and region. People experiencing homelessness were more likely to report having high school education or less (562 participants [11.7%] in the sample overall vs 82 [22.8%] among those experiencing homelessness; *P* < .001). Black participants were disproportionately represented among those experiencing homelessness compared with other racial groups (1339 participants [27.3%] of the sample overall vs 171 [44.1%] among those experiencing homelessness; *P* < .001). More than 40% of those experiencing homelessness reported lifetime injection drug use (164 participants [43.0%]) compared with 16.1% of those not experiencing homelessness (695 participants) (*P* < .001) (Table 1).

**Table 1.** Sociodemographic Characteristics of Gay, Bisexual, and Other Men Who Have Sex With Men Living With HIV Enrolled in the American Men’s Internet Survey, 2017 to 2023, by Current Experiences of Homelessness in the Past 12 Months

Characteristic	Individuals, No. (%)			*P* value
	Overall (N = 4911)	Never experienced homelessness in past 12 mo (n = 4352 [91.8%])	Experienced homelessness in past 12 mo (n = 388 [8.2%])	
Age, y				
15-24	202 (4.1)	178 (4.1)	18 (4.6)	.70
≥25	4709 (95.9)	4174 (95.9)	370 (95.4)	
Education				
High school education or less	562 (11.7)	392 (9.3)	82 (22.8)	<.001
Some college, associate’s degree, or more	4239 (88.3)	3813 (90.7)	278 (77.2)	
Race and ethnicity				
Black	1339 (27.3)	1145 (26.3)	171 (44.1)	
Hispanic	357 (7.3)	310 (7.1)	26 (6.7)	
White	1421 (28.9)	1262 (29.0)	49 (12.6)	
Other^a^	1794 (36.5)	1635 (37.6)	142 (36.6)	
Region				
Midwest	775 (15.8)	701 (16.1)	49 (12.6)	.10
Northeast	730 (14.9)	659 (15.1)	50 (12.9)	
South	2350 (47.9)	2061 (47.4)	209 (53.9)	
West	1048 (21.3)	923 (21.2)	80 (20.6)	
US dependent areas	7 (0.1)	7 (0.2)	0 (0.0)	
Pretax household income last year, US $				
≥40 000	2874 (55.5)	2725 (66.4)	63 (17.4)	<.001
<40 000	1737 (37.7)	1378 (33.6)	300 (82.6)	
Health insurance				
No insurance	4668 (95.1)	4176 (96.0)	330 (85.1)	<.001
Has insurance	243 (4.9)	176 (4.0)	58 (14.9)	
Injection drug use				
Never injected drugs	3982 (81.8)	3619 (83.9)	217 (57.0)	<.001
Ever injected drugs	883 (18.2)	695 (16.1)	164 (43.0)	
Year of recruitment				
2017	784 (16.0)	665 (15.3)	43 (11.1)	.001
2018	496 (10.1)	396 (9.1)	17 (4.4)	
2019	761 (15.5)	694 (15.9)	66 (17.0)	
2020	763 (15.5)	692 (15.9)	68 (17.5)	
2021	1060 (21.6)	961 (22.1)	97 (25.0)	
2022	498 (10.1)	440 (10.1)	56 (14.4)	
2023	549 (11.2)	504 (11.6)	41 (10.6)	
Current ART use				
Without	151 (3.1)	113 (2.6)	34 (8.8)	<.001
With	4760 (96.9)	4239 (97.4)	354 (91.2)	

Abbreviation: ART, antiretroviral therapy.

^a^ Other race and ethnicity included participants who self-reported as American Indian or Alaska Native, Asian, Native Hawaiian or Other Pacific Islander, and multiracial.

Figure, A shows the geographic distribution of the mean number of PLHIV experiencing homelessness by state between 2017 and 2023, dichotomized by the national median. Figure, B maps the mean state-level HOPWA funding across 2017 to 2022, dichotomized by the median. Some states with a higher mean number of homeless PLHIV—such as Connecticut and Rhode Island in the Northeast, and Arizona and Delaware in the South—were designated as having low levels of HOPWA funding. In the overall sample, homelessness was negatively associated with current ART use, adjusting for state-level Ryan White Part B funding (adjusted odds ratio [aOR], 0.28; 95% CI, 0.19-0.41).

**Figure.**
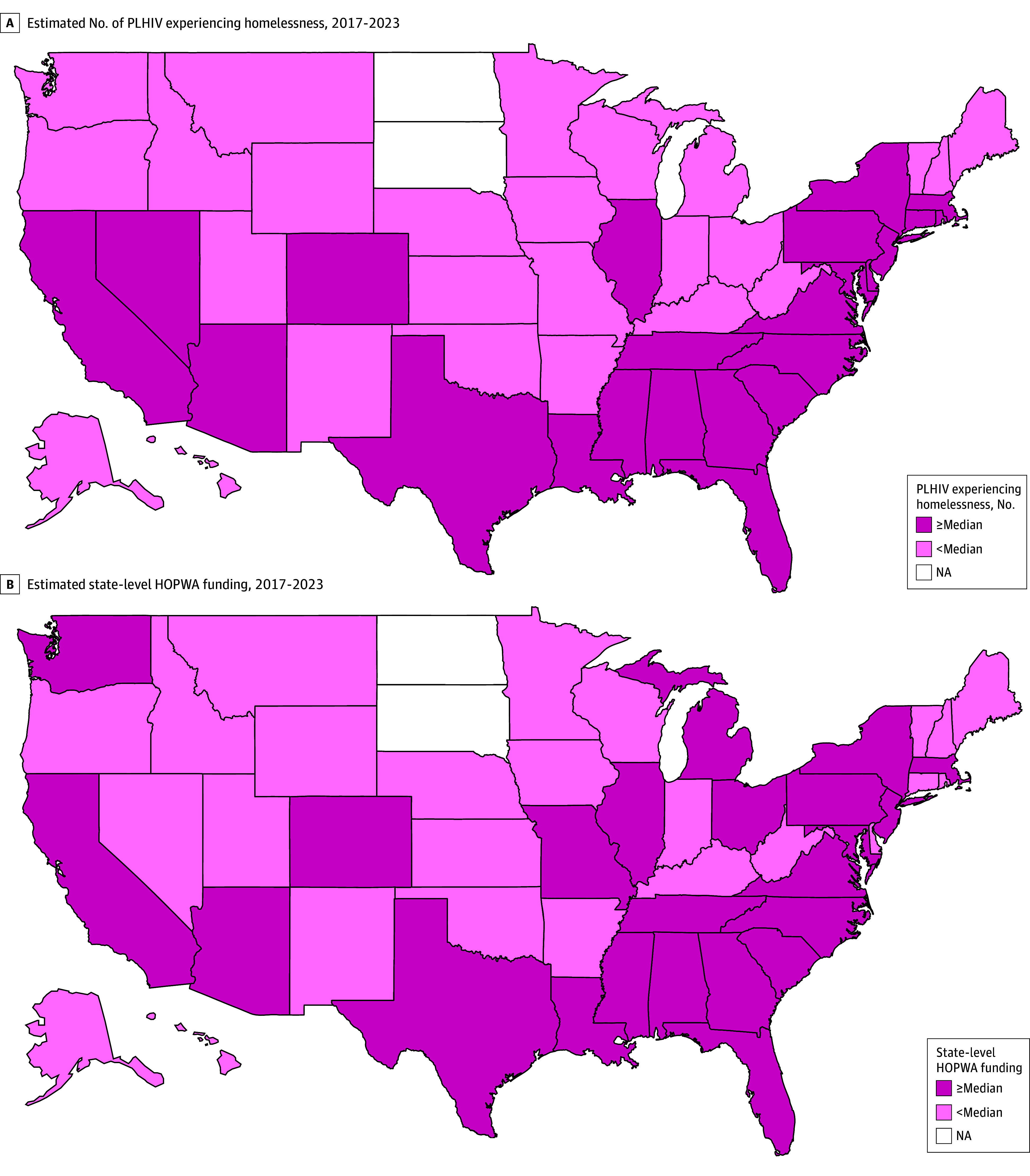
Maps Showing People Living With HIV (PLHIV) and Experiencing Homelessness and Housing Opportunities for Persons With HIV/AIDS (HOPWA) funding by State Between 2017 and 2023 A, Estimated median number of homeless individuals living with HIV by state between 2017 and 2023; B, Estimated median state-level HOPWA funding between 2017 and 2022. States were classified as having low or high HOPWA funding based on whether their per–person living with HIV experiencing homelessness funding level was at or below vs above the national median.

Accounting for individual and state-level characteristics, homelessness was negatively associated with current ART use overall (aOR, 0.51; 95% CI, 0.30-0.85; *P* < .001). When stratified by state-level HOPWA funding, homelessness remained negatively associated with current ART use (aOR, 0.43; 95% CI, 0.21-0.91) among participants from states that received at or below the median level of funding. Among participants from states that received above the median level of funding, there was no significant association between homelessness and current ART use (aOR, 0.61; 95% CI, 0.30-1.27).

Estimated probabilities from the adjusted mixed-effects logistic regression model found an estimated ART use probability of 91.5% (95% CI, 86.3%-94.9%) among nonhomeless participants and 84.6% (95% CI, 74.2%-91.3%) among those experiencing homelessness in the overall sample. When stratified by HOPWA funding, estimated probabilities from the adjusted mixed-effects logistic regression model found an estimated ART use probability of 90.6% (95% CI, 81.1%-95.6%) among nonhomeless participants and 80.7% (95% CI, 60.7%-91.9%) among those experiencing homelessness in the low funding stratum; in the high funding stratum, the adjusted model found an estimated ART use probability of 92.1% (95% CI, 84.7%-96.1%) among nonhomeless participants and 87.7% (95% CI, 74.4%-94.6%) among those experiencing homelessness (Table 2).

**Table 2.** Estimated Probabilities of Current Antiretroviral Therapy Use by Funding Strata and Experience of Homelessness Among Gay, Bisexual, and Other Men Who Have Sex With Men Living With HIV^a^

Stratum	Homeless status	Estimated probability, % (95% CI)
Overall	No	91.5 (86.3-94.9)
Overall	Yes	84.6 (74.2-91.3)
Low funding	No	90.6 (81.1-95.6)
Low funding	Yes	80.7 (60.7-91.9)
High funding	No	92.1 (84.7-96.1)
High funding	Yes	87.7 (74.4-94.6)

^a^ Data calculated using multivariable multilevel logistic regression model adjusted for potential confounders assessing the association between homelessness and use of antiretroviral therapy. Data were taken from the American Men’s Internet Survey, 2017 to 2023.

### Sensitivity Analyses

Using state-level HOPWA funding based on 2022 data alone, the results were similar compared with the original analyses (eTable 2 in Supplement 1). The results of the LPMs were directionally consistent with the mixed-effects logistic regression findings (eTable 3 in Supplement 1).

## Discussion

This cross-sectional study found that homelessness was associated with lower ART use among GBMSM in the US. Moreover, in the states that received a greater level of per-person HOPWA funding, this association was not observed. Some high-burden states received below-median HOPWA funding per PLHIV experiencing homelessness, reflecting a discrepancy between need and resources. These findings provide preliminary evidence that housing supports to address basic needs are associated with HIV treatment outcomes.

This study confirms that homelessness impedes ART use among GBMSM. Substandard housing conditions—including housing instability, inadequate structure, or poor-quality accommodations—have been linked with limited engagement in routine HIV care.^11^ In addition, lacking a permanent address may hinder communication with health care practitioners, leading to missed appointments and medication nonadherence.^11^ Before 2021, assistance programs such as the Ryan White AIDS Drug Assistance Program (ADAP) were poorly tailored for PLHIV who are unhoused and frequently lose ADAP eligibility if unable to provide recertification every 6 months.^31^ Similarly, Medicaid redeterminations often rely on mailed notices, which frequently go undelivered to people without stable housing and thus addresses, leading to coverage gaps and ART refill interruptions.^32^ Moreover, the chronic stress associated with securing basic housing may overshadow the urgency of HIV care, causing many to defer or abandon treatment altogether.^33^ Future interventions may benefit from integrated care models that colocate ART and health care services within settings accessible to unstably housed populations (eg, on-site pharmacies or deliveries to shelters or mobile pharmacies), with long-acting injectable ART that offer particular promise.^34,35^

The association between homelessness and lower ART use appears to be weaker in areas with higher per-person HOPWA funding. Because this study is observational, our findings should be interpreted as associations rather than causal effects, and residual confounding or selection bias may partly explain the observed associations. The stratified analyses found that the magnitude of the association between homelessness and ART differed across strata, suggesting potential contextual differences. HOPWA provides diverse housing supports to PLHIV experiencing homelessness, including tenant-based rental assistance, project-based rental assistance, short-term rent, mortgage and utility assistance, and master leasing arrangements, where subsidies are made directly to landlords, mortgage servicers, or utility providers.^36^ Such comprehensive housing assistance has been shown to significantly enhance housing affordability among low-income PLHIV, subsequently improving their HIV treatment adherence and overall health outcomes.^5^ Additionally, individuals enrolled in HOPWA programs receive regular case management to ensure consistent access to health care services, promote adherence to prescribed HIV medications, and facilitate access to additional resources such as ADAP and Patient Protection and Affordable Care Act benefits.^37^ Supporting these findings, a randomized trial conducted in Baltimore, Chicago, and Los Angeles demonstrated that HOPWA rental assistance significantly improved housing stability, health care utilization, and both mental and physical health outcomes.^38^ Similarly, in New York City—the largest HOPWA jurisdiction in the US—PLHIV enrolled in HOPWA were more likely to maintain primary health care engagement compared with their counterparts not receiving housing assistance, especially in high-poverty neighborhoods.^39^ Furthermore, a systematic review examining housing-first interventions in the US and Canada, a model closely aligned with HOPWA’s principles, found substantial health improvements for PLHIV, including a 22% reduction in viral load and a 41% decrease in emergency department visits, when compared with conventional treatment-first programs.^40^ Housing-first programs prioritize immediate access to permanent, low-barrier housing without requiring sobriety or treatment adherence as preconditions, whereas treatment-first models typically require individuals to achieve clinical stability or meet behavioral requirements before receiving permanent housing.^40^ Further research is needed to document the immediate and long-term impacts of this program.

Some high-burden states receive below-median HOPWA funding per homeless PLHIV, reflecting critical gaps between community need and resource allocation. Similarly, a national analysis using CDC’s Medical Monitoring Project data suggested that while nearly 28% of PLHIV reported needing housing assistance, approximately 40% of those needs remained unmet.^16^ Notably, the 2019 national HOPWA allocation provided was only sufficient to cover 1.24 months of rent per person annually.^16^ This discrepancy might be due to structural constraints in the HOPWA funding formula, which allocates funding primarily based on the number of PLHIV in a jurisdiction, without adequately accounting for how many of those individuals are unstably housed.^14,15^ The HOPWA funding formula also overlooked accountability for local poverty rates among PLHIV, who face higher economic marginalization than the general population.^41^ Additionally, using the PIT count, which occurs on a single night, typically undercounts those couch-surfing or doubled-up and unsheltered individuals, such as those encamped outside official zones.^29^ These results suggest that policymakers should supplement formula-based allocations with targeted resources for states where PLHIV experiencing homelessness are likely undercounted to ensure HOPWA aligns with community needs.

### Limitations

This study has limitations. The cross-sectional nature of AMIS restricts our ability to infer causality in the associations assessed. Furthermore, collider bias is possible if ART use and homelessness share common causes that also affect survey participation. Those who were able to complete the survey may have a fundamentally different experience as it relates to homelessness and ART use than those who were not able to enter the study and complete the survey. Although imperfect, we adjusted for several key sociodemographic factors that may also be related to participation, which may partially mitigate this concern. Additionally, AMIS uses online, convenience-based recruitment and self-reported HIV outcomes; people experiencing homelessness may therefore be underrepresented, and selection, coverage, and misclassification biases may limit generalizability and bias estimated associations. At the same time, the online recruitment approach enables broad geographic reach and a larger sample size than would be feasible with many venue-based methods. Moreover, the dichotomization of homelessness may have biased associations by grouping individuals with substantially different levels of housing vulnerability into a single category. Finally, because direct state-level counts of people experiencing homelessness and living with HIV were unavailable, we relied on proxy measures and multiyear means to characterize the relative HOPWA funding environment for median-based stratification across states. This approach may obscure year-to-year variation, reduce statistical power, and limit our ability to detect potential dose-response associations.

## Conclusions

Higher per-person HOPWA funding may mitigate homelessness-related barriers to ART use among GBMSM living with HIV. These results underscore the disproportionate disparities in ART use among GBMSM living with HIV experiencing homelessness in the US. Future interventions may benefit from integrated care models that colocate ART and health care services within settings accessible to under- or unstably housed populations, while employing tailored strategies to help navigate specific challenges in sustained ART use. While homelessness was significantly associated with lower ART use, this association appears to be attenuated in states with higher per-person HOPWA funding, underscoring the positive outcomes of housing assistance in decreasing HIV-related morbidity but also ultimately supporting HIV epidemic control.

## Notes

Supplement 1. eTable 1. State-Level HOPWA Funding per Person and State-Level Ryan White Funding eTable 2. Sensitivity Analysis of the Multivariable Multilevel Logistic Regression Model Between Homelessness and ART Use Using State-Level HOPWA Funding Defined by 2022 Data Alone eTable 3. Prevalence Difference Estimates From Linear Probability Models eTable 4. Distribution of ART Use by Homelessness Status, Overall and Stratified by Funding

Supplement 2. Data Sharing Statement
