# Supplementary material for: Homelessness and HIV Treatment Among Men Who Have Sex With Men Across US Funding Contexts
Source: JAMA Netw Open. 2026 May 19;9(5):e2613609. doi: 10.1001/jamanetworkopen.2026.13609 (PMC13187872; doi:10.1001/jamanetworkopen.2026.13609)
Supplement: Supplement 1. — eTable 1. State-Level HOPWA Funding per Person and State-Level Ryan White Funding eTable 2. Sensitivity Analysis of the Multivariable Multilevel Logistic Regression Model Between Homelessness and ART Use Using State-Level HOPWA Funding Defined by 2022 Data Alone eTable 3. Prevalence Difference Estimates From Linear Probability Models eTable 4. Distribution of ART Use by Homelessness Status, Overall and Stratified by Funding [file jamanetwopen-e2613609-s001.pdf]

Supplemental Online Content

Mi Y, Baral SD, Voet KA, et al. Homelessness and HIV treatment among men who have sex with men across US funding contexts. *JAMA Netw Open*. 2026;9(5):e2613609. doi:10.1001/jamanetworkopen.2026.13609

eTable 1 State-Level HOPWA Funding per Person and State-Level Ryan White Funding
eTable 2. Sensitivity Analysis of the Multivariable Multilevel Logistic Regression Model Between Homelessness and ART Use Using State-Level HOPWA Funding Defined by 2022 Data Alone
eTable 3 Prevalence Difference Estimates From Linear Probability Models
eTable 4 Distribution of ART Use by Homelessness Status, Overall and Stratified by Funding

This supplemental material has been provided by the authors to give readers additional information about their work.

© 2026 Mi Y et al. *JAMA Network Open*.

eTable 1 State-Level HOPWA Funding per Person and State-Level Ryan White Funding

State*	Mean HOPWA funding each year (USD)	Mean number of homeless individuals seen by state	Mean number of homeless individuals seen by state living with HIV	Level of state-level average number of homeless individuals seen by state living with HIV (high: above the median; low, at or below the median)	Mean Ryan White funding each year (USD)	State-level HOPWA funding per person (USD)	Level of state-level HOPWA funding per person (high: above the median; low, at or below the median)
AK	892274.80	2086.00	150.00	low	1181901.00	285162.93	high
AL	5634834.29	3350.00	400.84	high	19605067.00	419628.80	high
AR	1640332.14	2496.00	287.04	low	8132230.00	228952.19	low
AZ	5559063.57	10435.00	345.64	high	24613832.00	154129.29	low
CA	53378667.60	140674.00	465.40	high	146373830.00	81531.89	low
CO	3711416.29	10663.00	305.22	high	16194028.00	114037.39	low
CT	3673450.00	3120.00	363.10	high	18076488.00	324259.93	high
DC	11713115.00	5960.00	1979.74	high	14718250.00	99269.99	low
DE	1517932.57	1336.00	465.34	high	5182622.00	244160.63	low
FL	44996594.70	28124.00	701.32	high	126440830.00	228132.07	low
GA	26458819.30	9831.00	766.74	high	73294839.00	351014.17	high
HI	2311948.57	5900.00	217.18	low	3408765.00	180428.99	low
IA	737749.71	2582.00	130.32	low	10298406.00	219251.08	low
ID	1416713.00	2123.00	109.08	low	3357252.00	611768.00	high

© 2026 Mi Y et al. *JAMA Network Open*.

IL	16438589.70	10170.00	380.74	high	50352902.00	424536.56	high
IN	3623902.57	5462.00	251.18	low	22741446.00	264143.40	high
KS	622922.86	2310.00	164.68	low	5413312.00	163750.05	low
KY	2811746.29	3871.00	250.42	low	10400018.00	290057.40	high
LA	9061984.57	3593.00	647.46	high	23690910.00	389540.99	high
MA	8509473.60	17687.00	376.52	high	20632685.00	127779.29	low
MD	11936548.70	6154.00	708.28	high	34110979.00	273852.24	high
ME	1599782.00	2819.00	157.40	low	1789499.00	360546.27	high
MI	6289494.43	8368.00	226.34	low	18719538.00	332072.38	high
MN	2943818.14	7702.00	211.54	low	8581972.00	180682.03	low
MO	6320924.86	6036.00	280.72	low	13564474.00	373042.27	high
MS	3876391.00	1131.00	465.66	high	14236353.00	736030.88	high
MT	1536776.60	1629.00	80.54	low	1351909.00	1171326.66	high
NC	9386655.00	9030.00	432.94	high	43488143.00	240101.79	low
NE	631815.57	2333.00	166.40	low	5220702.00	162750.47	low
NH	1625506.83	1645.00	120.52	low	1418716.00	819905.45	high
NJ	13715470.00	9105.00	502.76	high	40481053.00	299619.47	high
NM	1437728.43	2965.00	248.10	low	4435016.00	195445.38	low
NV	2645868.29	7546.00	505.42	high	10761757.00	69374.36	low
NY	54300023.40	88723.00	783.60	high	132922898.00	78103.30	low
OH	6780049.86	10186.00	277.28	low	25020400.00	240054.95	low
OK	2071443.14	3863.00	249.66	low	8838282.00	214782.72	low

© 2026 Mi Y et al. *JAMA Network Open*.

OR	3625114.43	15158.00	227.22	low	6586378.00	105252.70	low
PA	13400461.60	12830.00	360.90	high	37561096.00	289405.13	high
PR	8579566.43	2370.00	592.58	high	28199587.00	610899.83	high
RI	1873149.14	1299.00	308.44	high	5380318.00	467511.73	high
SC	5741043.29	3882.00	476.06	high	26961772.00	310651.60	high
TN	7661620.86	8217.00	367.62	high	26053840.00	253634.45	low
TX	31983752.90	24434.00	490.44	high	123215193.00	266900.24	high
UT	910765.43	3209.00	149.72	low	6460998.00	189564.50	low
VA	4802293.00	6126.00	374.84	high	26620415.00	209134.52	low
VI	1514229.67	298.00	NA	NA	NA	NA	NA
VT	1066133.40	1912.00	134.82	low	1196820.00	413589.34	high
WA	4947210.71	21811.00	250.64	low	13749395.00	90497.06	low
WI	2898418.14	4694.00	152.74	low	8650897.00	404264.09	high
WV	593886.00	1317.00	182.18	low	4492446.00	247523.60	low
WY	403427.00	608.00	83.72	low	748222.00	792560.02	high

*There is no HOPWA funding data retrieved from the U.S. Department of Housing and Urban Development database for AS, GU, MP, ND, or SD; there is no data on the average adult HIV prevalence estimates (per 100,000 population) for each year 2018–2022 from the Centers for Disease Control and Prevention (CDC) HIV Surveillance Report for VI.

© 2026 Mi Y et al. *JAMA Network Open*.

eTable 2. Sensitivity Analysis of the Multivariable Multilevel Logistic Regression Model Between Homelessness and ART Use Using State-Level HOPWA Funding Defined by 2022 Data Alone

Experienced homelessness in past 12 months (ref: Never homeless in past 12 months)	State-level HOPWA funding+<br>At or below median level of funding states aOR (95% CI)	 Above median level of funding states aOR (95% CI)
Sensitivity analysis using state-level cutoffs based on 2022 HOPWA data alone)	**0.34 (0.17, 0.68)**	0.85 (0.38, 1.90)

eTable 3 Prevalence Difference Estimates From Linear Probability Models

Analysis	Stratification	Model	Prevalence difference (pp*)	95% CI (pp)	P value
**Overall**	Overall	Adjusted	**−3.52**	−6.57 to −0.47	0.024
**Stratified**	Low funding	Adjusted	−3.49	−7.14 to +0.15	0.060
	High funding	Adjusted	−3.40	−8.34 to +1.55	0.178

*pp indicates percentage points; estimates represent prevalence differences in current ART use.

eTable 4 Distribution of ART Use by Homelessness Status, Overall and Stratified by Funding

Stratum	Homeless	ART = No	ART = Yes	ART use (%)
Overall	No	113	4,238*	**97.4**
Overall	Yes	34	354	**91.2**
Low funding	No	60	2,363	**97.5**
Low funding	Yes	15	183	**92.4**
High funding	No	53	1,865	**97.2**
High funding	Yes	19	171	**90.0**

* 10 participants from states without HOPWA funding data or CDC HIV data were dropped from stratified analyses.

© 2026 Mi Y et al. *JAMA Network Open*.
