# Supplementary material for: Homelessness and HIV Treatment Among Men Who Have Sex With Men Across US Funding Contexts
Source: JAMA Netw Open. 2026 May 19;9(5):e2613609. doi: 10.1001/jamanetworkopen.2026.13609 (PMC13187872; doi:10.1001/jamanetworkopen.2026.13609)
Supplement: Supplement 2. — Data Sharing Statement [file jamanetwopen-e2613609-s002.pdf]

Data Sharing Statement

Mi. Homelessness and HIV Treatment Among Men Who Have Sex With Men Across US Funding Contexts. *JAMA Netw Open*. Published May 19, 2026.
doi:10.1001/jamanetworkopen.2026.13609

Data
Data available: No

Additional Information
Explanation for why data not available: Data will be made available on request.
